# Laparoscopic Versus Open Total Gastrectomy for Advanced Gastric Cancer: A Multicenter, Propensity Score-Matched Cohort Study in China

**DOI:** 10.3389/fonc.2021.780398

**Published:** 2021-12-13

**Authors:** Xingyu Feng, Xin Chen, Zaisheng Ye, Wenjun Xiong, Xueqing Yao, Wei Wang, Junjiang Wang, Luchuan Chen, Yong Li

**Affiliations:** ^1^ Department of Gastrointestinal Surgery, Guangdong Provincial People’s Hospital, Guangdong Academy of Medical Sciences, Guangzhou, China; ^2^ The Second School of Clinical Medicine, Southern Medical University, Guangzhou, China; ^3^ Shantou University Medical College, Shantou, China; ^4^ Department of Gastrointestinal Surgical Oncology, Fujian Cancer Hospital and Fujian Medical University Cancer Hospital, Fuzhou, China; ^5^ Department of Gastrointestinal Surgery, Guangdong Provincial Hospital of Chinese Medicine, The Second Affiliated Hospital of Guangzhou University of Chinese Medicine, Guangzhou, China

**Keywords:** advanced gastric cancer, laparoscopic total gastrectomy with D2 lymph node dissection for gastric cancer, open total gastrectomy, multicenter cohort study, propensity score (PS) matching (PSM)

## Abstract

**Background:**

Given the great technical difficulty and procedural complexity of laparoscopic total gastrectomy (LTG), the technical and oncologic safety of LTG versus open total gastrectomy (OTG) in the field of advanced gastric cancer (AGC) is yet undetermined.

**Objective:**

This multicenter cohort study aimed to compare the surgical and oncological outcomes of LTG with those of OTG in AGC patients.

**Patients and Methods:**

In total, 588 patients from 3 centers who underwent primary total gastrectomy with D2 lymphadenectomy, by well-trained surgeons with adequate experience, for pathologically confirmed locally AGC (T2N0–3, T3N0–3, or T4N0–3) between January 1, 2011, and December 31, 2015, were identified, and their clinical data were collected from three participating centers. After 1:1 propensity score matching (PSM), 450 cases (LTG, n = 225; OTG, n = 225) were eligible and assessed.

**Results:**

No significant difference in the number of retrieved lymph nodes, 5-year disease-free survival (DFS) rates, or 5-year overall survival (OS) rates between both surgical groups were observed. Although LTG had significantly longer surgical time (262 vs. 180 min, *p* < 0.001), LTG was associated with fewer postoperative complications [relative risk (RR) 0.583, 95% CI 0.353–0.960, *p* = 0.047), less intraoperative bleeding (120 vs. 200 ml, *p* < 0.001), longer proximal margin resection (3 vs. 2 cm, *p* < 0.001), and shorter postoperative hospitalization (11 vs. 13 days, *p* < 0.001). The mortality rate was comparable in both groups.

**Conclusions:**

LTG was not inferior to OTG in terms of survival outcomes and was associated with shorter surgical and postoperative hospitalization time and fewer postoperative complications, suggesting LTG with D2 lymphadenectomy as an important alternative to OTG for patients with AGC, but to be carried out in highly experienced centers.

## Introduction

Gastric cancer is still one of the most common and lethal cancers worldwide and in China (1, 2). Gastrectomy remains the primary and most effective treatment for invasive gastric cancer, although some superficial cancers can be resected endoscopically (3). Despite a decline in the incidence of distal gastric cancer, the incidence of proximal gastric cancer has been significantly increasing (4). Currently, total gastrectomy remains the preferred treatment for proximal gastric cancer, while proximal gastrectomy is usually reserved for selected patients, as it has been shown to offer nutritional benefits to these patients (5). Either laparoscopic or open gastrectomy can be performed, but both approaches are technically demanding and are recommended to be performed by well-trained surgeons at institutions with extensive experience in gastrectomy.

In regard to early gastric cancer (EGC), only two multicenter randomized clinical trials (RCTs), KLASS03 (6) and CLASS02 (7), have established the feasibility and safety of laparoscopic total gastrectomy (LTG), and studies on the oncological safety of LTG are still ongoing. However, the safety of LTG for advanced gastric cancer (AGC) remains uncertain in the absence of high-level clinical evidence from RCTs. Although satisfactory surgical and oncological outcomes from LTG for AGC have been reported by several studies (8–15), they are limited due to almost exclusively being retrospective studies of small sample sizes from single centers. Until now, there have been only two retrospective multicenter cohort studies based on Japanese nationwide databases, which evaluated the effects of LTG versus open total gastrectomy (OTG) on the surgical outcomes among patients with AGC, but they lacked long-term oncological outcome results, which are clinically necessary to fully support LTG as an oncologically safe alternative to OTG for AGC (16, 17).

Thus, this multicenter comparative study was conducted to evaluate both the technical and oncological safety of LTG versus OTG in the field of AGC by comparing the surgical and oncological outcomes of both approaches.

## Patients and Methods

### Patients and Study Protocol

Gastric cancer cases with pathologically confirmed locally advanced gastric adenocarcinoma (pT2N0–3, pT3N0–3, or pT4N0–3) who underwent primary LTG or OTG with D2 lymphadenectomy from January 1, 2011, to December 31, 2015, at 3 centers were enrolled (292 cases from Fujian Cancer Hospital and Fujian Medical University Cancer Hospital, 118 cases from Guangdong Provincial Hospital of Chinese Medicine and the Second Affiliated Hospital of Guangzhou University of Chinese Medicine, and 178 cases from Guangdong Provincial People’s Hospital and Guangdong Academy of Medical Sciences). The 8th edition of the American Joint Committee on Cancer/Union for International Cancer Control (AJCC/UICC) TNM staging classification was used to update the pathologic staging of all cases.

To rule out metastases, a CT scan of the abdomen for preoperative staging was routinely conducted. Upper endoscopy was also used to assess the extent of locoregional tumors as well as the anatomy of the esophagus and stomach, which is important for surgeons to choose the type of reconstruction. Standard surgical procedures of total gastrectomy with D2 lymphadenectomy were performed as described in the Japanese gastric cancer treatment guidelines 2014 (ver. 4) (18) to guarantee the consistency of the operation and the quality of the study. Surgical margins were evaluated by frozen resection, as indicated, and when no margin invasion was histologically confirmed, surgeons continued reconstruction for restoring gastrointestinal continuity.

The study protocol was unanimously approved by the ethical committee of each participating center, and informed consent was obtained from each patient before treatment. Eligibility criteria were as follows: 1) clinical stage IB–IIIC gastric cancer and 2) tumors located in the upper or middle third of the stomach. Exclusion criteria included 1) concurrence of other primary malignancy, 2) tumors down-staged from stage IV by neoadjuvant therapy, and 3) emergency surgery cases. The demographic and clinicopathological data of consecutive eligible patients were retrospectively collected.

The primary endpoints of this study included overall survival (OS) and disease-free survival (DFS). OS was defined as the interval between the date of surgery and any cause of death or the last follow-up, while DFS was defined as the interval between the date of surgery and any confirmed recurrence or death from any cause. The secondary endpoints were morbidity and mortality within 30 days following the gastrectomy. Surgical time, estimated blood loss, the number of retrieved lymph nodes, time to first liquid intake, time to ambulation, postoperative hospital stay, length and status of proximal resection margin, and distal resection margin length were also retrieved and evaluated.

### Statistical Analysis

Case matching was performed using the propensity score of 7 factors: sex, age, body mass index (BMI), tumor size, tumor histology, pathologic T stage, and pathologic TNM stage. A 1:1 nearest-neighbor propensity score matching (PSM) without replacement was used, with the caliper set at 0.2 (19). Patients who were outside the caliper or unmatched were excluded. Only matched-paired patients were involved in the statistical analyses for primary and secondary endpoints. The Kaplan–Meier survival curves were calculated for the LTG group and OTG group and were compared by log-rank test. We adopted the Mann–Whitney U test to analyze continuous variables, which were presented as medians with interquartile ranges (IQRs). The chi-square test was adopted to analyze categorical variables. Univariate and multivariate analyses with the Cox regression model were performed to identify independent risk factors for DFS and OS. All 2-sided *p*-values <0.05 were considered statistically significant. The SPSS software (ver. 25.0; SPSS, Inc., Chicago, IL, USA) was used to conduct all statistical analyses.

## Results

A total of 322 cases underwent LTG, while the remaining 266 patients underwent OTG. After PSM, 225 patients underwent LTG and OTG (220 cases from Fujian Cancer Hospital and Fujian Medical University Cancer Hospital, 90 cases from Guangdong Provincial Hospital of Chinese Medicine and the Second Affiliated Hospital of Guangzhou University of Chinese Medicine, and 140 cases from Guangdong Provincial People’s Hospital and Guangdong Academy of Medical Sciences). **Table 1** summarizes the baseline clinical characteristics of the patients before and after PSM. The demographic factors and baseline oncological characteristics of the matched cases, compared with the overall cohort, were well balanced between the LTG and the OTG groups, except that patients with deeper pathologic tumor depth and larger tumor size tended to undergo OTG, while patients with poorer histologic differentiation tended to receive LTG.

**Table 1:** Baseline characteristics before and after propensity score matching.

	Overall cohort			After matching		
Characteristic	LTG (n = 322)	OTG (n = 266)	*p*	LTG (n = 225)	OTG (n = 225)	*p*
**Sex**			0.068			0.351
** Male**	218 (67.7%)	199 (74.8%)		154 (68.4%)	164 (72.9%)	
** Female**	104 (32.3%)	67 (25.2%)		71 (31.6%)	61 (27.1%)	
**Age, years**	59 (50–66)	61 (53–67)	0.171	59 (50–67)	61 (53–67)	0.297
**BMI, kg/m^2^**	22.1 (20.1–24.4)	22.1 (20.3–24.1)	0.841	21.9 (19.9–24.0)	22.2 (20.3–24.2)	0.164
**Tumor size, cm**	4 (3–5)	5 (3–7)	<0.001	4 (3–5)	5 (3–6.5)	<0.001
**Histology**			<0.001			0.001
** Differentiated**	87 (27%)	115 (43.2%)		60 (26.7%)	93 (41.3%)	
** Undifferentiated**	235 (73%)	151 (56.8%)		165 (73.3%)	132 (58.7%)	
** Metastatic lymph node**	3 (0–7)	5 (1–10)	0.001	4 (1–9)	4 (1–8)	0.943
**Pathologic T stage**			<0.001			<0.001
** T2**	38 (11.8%)	34 (12.8%)		19 (8.4%)	33 (14.7)	
** T3**	129 (40.1%)	18 (6.8%)		82 (36.4%)	17 (7.6%)	
** T4a**	152 (47.2%)	211 (79.3%)		121 (53.8%)	172 (76.4%)	
** T4b**	3 (0.9%)	3 (1.1%)		3 (1.3%)	3 (1.3%)	
**Pathologic N stage**			0.001			0.770
** N0**	93 (28.9%)	52 (19.5%)		50 (22.2%)	45 (20.0%)	
** N1**	78 (24.2%)	56 (21.1%)		48 (21.3%)	53 (23.6%)	
** N2**	65 (20.2%)	63 (23.7%)		51 (22.7%)	61 (27.1%)	
** N3**	86 (26.7%)	95 (35.7%)		76 (33.8%)	66 (29.3%)	
**Pathologic M stage**			–			–
** M0**	322 (100%)	266 (100%)		225 (100%)	225 (100%)	
** M1**	0 (0%)	0 (0%)		0 (0%)	0 (0%)	
**Pathologic TNM stage**			<0.001			0.057
** IB**	20 (6.2%)	19 (7.1%)		8 (3.6%)	18 (8.0%)	
** IIA**	51 (15.8%)	11 (4.1%)		24 (10.7%)	11 (4.9%)	
** IIB**	72 (22.4%)	37 (13.9%)		47 (20.9%)	31 (13.8%)	
** IIIA**	60 (18.6%)	42 (15.8%)		42 (18.7%)	39 (17.3%)	
** IIIB**	63 (19.6%)	64 (24.1%)		53 (23.6%)	61 (27.1%)	
** IIIC**	56 (17.4%)	93 (35.0%)		51 (22.7%)	65 (28.9%)	

LTG, laparoscopic total gastrectomy; OTG, open total gastrectomy; PSM, propensity score matching; BMI, body mass index.

The surgical outcomes are summarized in **Tables 2**, **3**. The LTG group had a significantly lower estimated blood loss (120 vs. 200 ml, *p* < 0.001), a longer length of proximal resection margin (3 vs. 2 cm, *p* < 0.001), and significantly shorter postoperative hospitalization (11 vs. 13 days, *p* < 0.001) than the OTG group. However, LTG was associated with significantly longer surgical time (262 vs. 180 min, *p* < 0.001). There was no significant difference in the status of proximal or distal resection margin, the number of retrieved lymph nodes, time to first liquid, or time to ambulation found between the two groups. It is important to note that according to the National Comprehensive Cancer Network (NCCN) Clinical Practice Guidelines in Oncology for Gastric Cancer (version 2. 2013), retrieval of a minimum of 15 lymph nodes is essential to ensure accurate staging of the patient (20), and in this study, we found that both the LTG and OTG approaches could meet this minimum requirement (95.6% vs. 96.0%, *p* > 0.999).

**Table 2:** Comparison of the surgical outcomes of the two surgical approaches.

Outcome	LTG (n = 225)	OTG (n = 225)	*p*
**Surgical time, minutes**	262 (210–312)	180 (160–208)	<0.001
**Estimated blood loss, ml**	120 (80–200)	200 (120–300)	<0.001
**Proximal resection margin, cm**	3 (2–5)	2 (1.5–2.5)	<0.001
**Proximal resection margin status**			0.372
** Negative**	224 (99.6%)	221 (98.2%)	
** Positive**	1 (0.04%)	4 (1.8%)	
**Distal resection margin status**			>0.999
** Negative**	225 (100%)	225 (100%)	
** Positive**	0 (0%)	0 (0%)	
** Number of retrieved LN**	29 (22–40.5)	31 (23.5–41)	0.200
**LN retrieval**			>0.999
** ≥15**	215 (95.6%)	216 (96.0%)	
** <15**	10 (4.4%)	9 (4.0%)	
**Time to first liquid intake, days**	3 (1–5)	2 (2–3)	0.443
**Time to ambulation, days**	2 (1–3)	2 (1–3)	0.258
**Postoperative hospital stay, days**	11 (9–13.5)	13 (11–15)	<0.001

LTG, laparoscopic total gastrectomy; OTG, open total gastrectomy; LN, lymph node.

**Table 3:** Postoperative morbidities and mortality between the LTG and OTG groups.

	LTG (n = 225)	OTG (n = 225)	RR	95% CI	*p*
**Mortality**	0	1 (0.4%)	0.000	0.000–3.828	>0.999
**Postoperative complication**	21 (9.3%)	36 (16.0%)	0.583	0.353–0.960	0.047
** Anastomotic leakage**	7 (3.1%)	9 (4.0%)	0.778	0.305–1.981	0.800
** Anastomotic stenosis**	2 (0.9%)	0	Infinity	0.524–Infinity	0.499
** Intraluminal bleeding**	2 (0.9%)	5 (2.2%)	0.400	0.090–1.766	0.449
** Intra-abdominal bleeding**	3 (1.3%)	1 (0.4%)	3.000	0.433–20.860	0.623
** Ileus**	6 (2.7%)	2 (0.9%)	3.000	0.701–12.910	0.285
** Gastroparesis**	0	0	–	–	>0.999
** Wound problem**	3 (1.3%)	2 (0.9%)	1.500	0.302–7.455	>0.999
** Intra-abdominal abscess**	7 (3.1%)	22 (9.8%)	0.318	0.141–0.710	0.006
** Lymphatic leakage**	1 (0.4%)	1 (0.4%)	1.000	0.105–9.544	>0.999
** Chylous leakage**	1 (0.4%)	4 (1.8%)	0.250	0.038–1.647	0.372
** Urinary retention**	1 (0.4%)	0	Infinity	0.261–Infinity	>0.999
**Clavien–Dindo classification**					
** Grade I**	9 (4.0%)	14 (6.2%)	0.643	0.290–1.422	0.392
** Grade II**	8 (3.6%)	14 (6.2%)	0.571	0.250–1.301	0.274
** Grade III**	2 (0.9%)	6 (2.7%)	0.333	0.077–1.426	0.285
** Grade IV**	2 (0.9%)	1 (0.4%)	2.000	0.264–15.210	>0.999
** Grade V**	0	1 (0.4%)	0.000	0.000–3.828	>0.999
** Clavien–Dindo grade ≥ III**	4 (1.8%)	8 (3.6%)	0.500	0.162–1.537	0.381

LTG, laparoscopic total gastrectomy; OTG, open total gastrectomy; RR, relative risk.

LTG did not significantly differentiate in the mortality rate from OTG (RR 0.000, 95% CI 0.000–3.828, *p* > 0.999). Compared with OTG, LTG had significantly fewer overall postoperative complications (RR 0.583, 95% CI 0.353–0.960, *p* = 0.047) and reduced incidence of intra-abdominal abscess (RR 0.318, 95% CI 0.141–0.710, *p* = 0.006), and the risk of anastomotic leak was comparable in both techniques (RR 0.778, 95% CI 0.305–1.981, *p* = 0.800). Further, major surgical complications (Clavien–Dindo grade ≥III) were comparable in both techniques (RR 0.500, 95% CI 0.162–1.537, *p* = 0.381).

**Figure 1:**
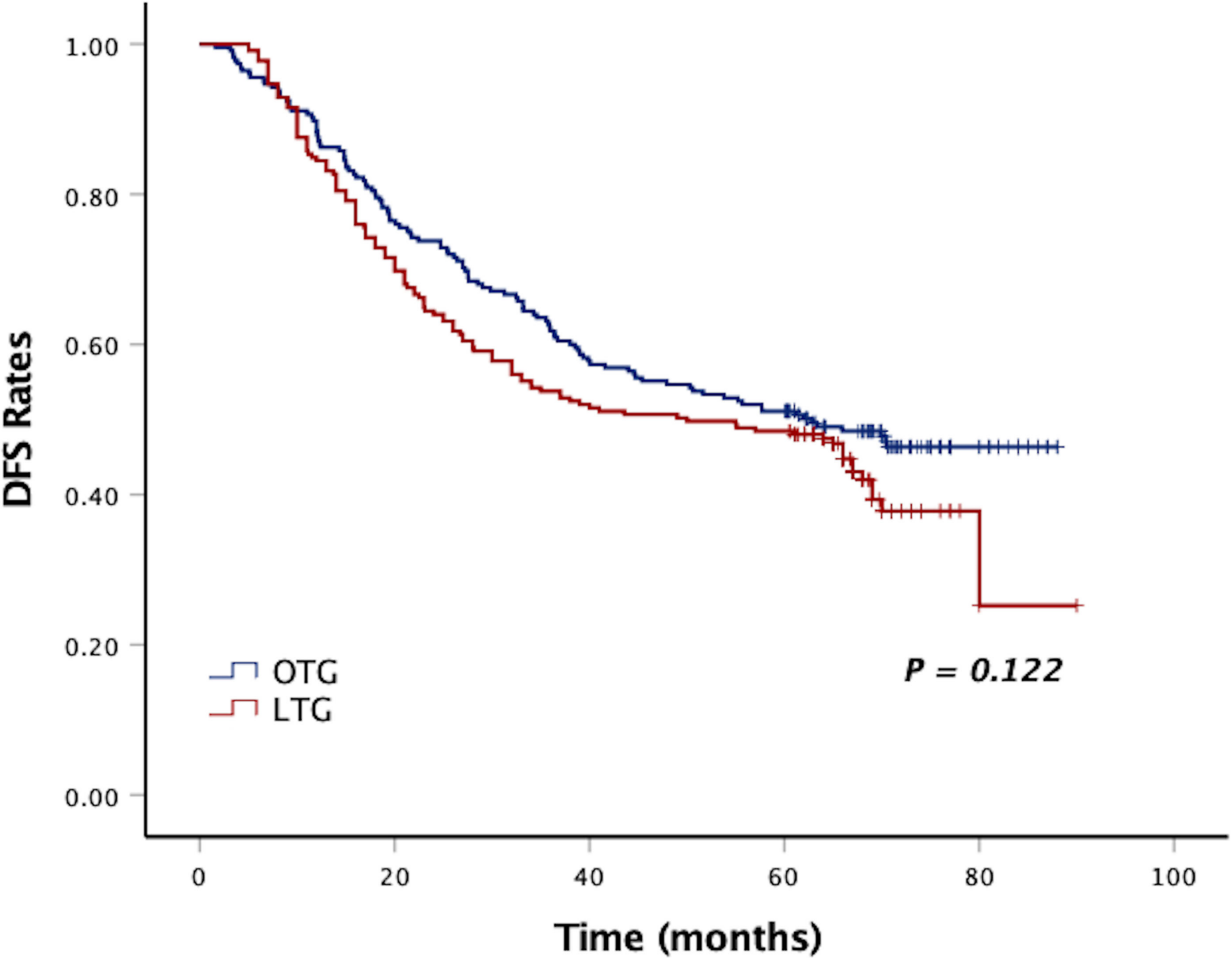
Kaplan–Meier curves of DFS between the LTG and OTG groups. DFS, disease-free survival; LTG, laparoscopic total gastrectomy; OTG, open total gastrectomy.

**Figure 2:**
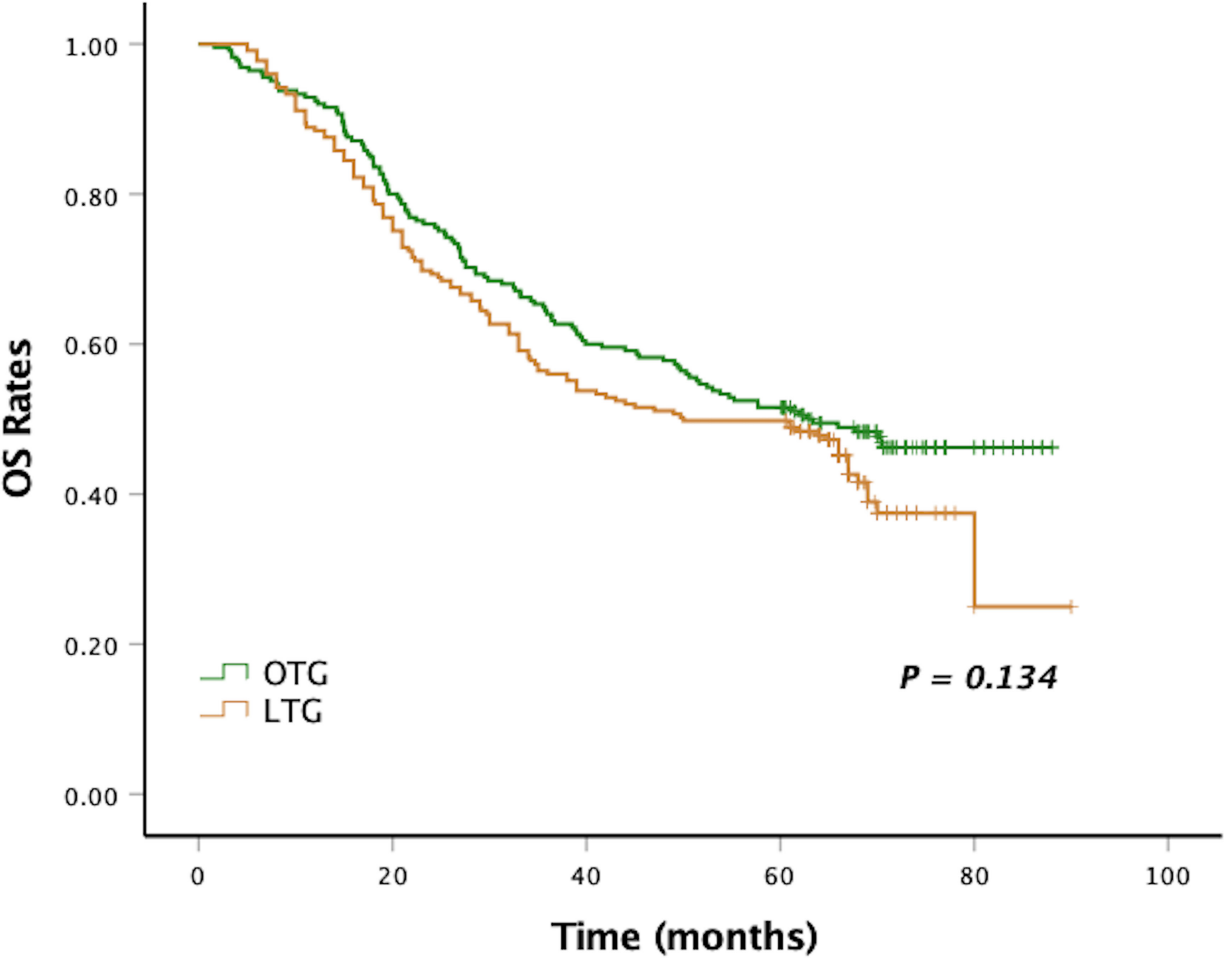
Kaplan–Meier curves of OS between the LTG and the OTG groups. OS, overall survival; LTG, laparoscopic total gastrectomy; OTG, open total gastrectomy.

After a median follow-up period of 58 months (range, 2–90 months), the 5-year DFS and 5-year OS of LTG were statistically similar to those of OTG (5-year DFS: 48.0% vs. 50.6%, *p* = 0.122, **Figure 1**, and 5-year OS: 48.9% vs. 51.1%, *p* = 0.134, **Figure 2**). Univariate analyses of the risk factors for oncological outcomes showed that tumor size, tumor histology, number of metastatic lymph nodes, pT stage, pN stage, and pTNM stage were associated with DFS and OS (**Tables 4**, **5**). Multivariate analyses identified pT stage and pTNM stage as independent predictors for DFS and OS. Surgical techniques failed to be an independent risk factor for DFS or OS (HR 1.218, 95% CI 0.948–1.565, *p* = 0.123; HR 1.211, 95% CI 0.942–1.556, *p* = 0.135).

**Table 4:** Univariate and multivariate analyses of the risk factors for overall survival.

	Univariate			Multivariate		
	HR	95% CI	*p*	HR	95% CI	*p*
**Sex**			0.060			
Male	1					
Female	1.290	0.989–1.683				
**Age, years**			0.262			
<60	1					
≥60	1.154	0.899–1.483				
**BMI, kg/m^2^**			0.621			
<24	1					
≥24	1.072	0.814–1.412				
**Tumor size, cm**			< 0.001			
<5	1					
≥5	1.632	1.267–2.100				
**Histology**			0.013			
Differentiated	1					
Undifferentiated	1.413	1.074–1.857				
**Metastatic lymph node**			<0.001			
<3	1					
≥3	2.757	2.068–3.675				
**Pathologic T stage**			<0.001			0.006
T2	1			1		
T3	1.490	0.805–2.757	0.204	0.518	0.249–1.081	0.080
T4a	3.330	1.934–5.733	<0.001	0.958	0.468–1.958	0.906
T4b	9.355	3.565–24.549	<0.001	1.813	0.612–5.367	0.283
**Pathologic N stage**			<0.001			
N0	1					
N1	2.210	1.346–3.628	0.002			
N2	3.901	2.437–6.245	<0.001			
N3	5.024	3.191–7.909	<0.001			
**Pathologic TNM stage**			<0.001			<0.001
IB	1			1		
IIA	1.882	0.579–6.113	0.293	2.400	0.680–8.467	0.173
IIB	2.359	0.823–6.760	0.110	2.614	0.781–8.752	0.119
IIIA	4.264	1.530–11.883	0.006	4.804	1.420–16.249	0.012
IIIB	6.290	2.298–17.218	<0.001	7.372	2.159–25.170	0.001
IIIC	10.208	3.746–27.819	<0.001	10.619	3.100–36.375	<0.001
**Retrieved lymph node**			0.051			
<28	1					
≥28	1.293	0.999–1.675				
**Surgical approach**			0.135			
Open	1					
Laparoscopic	1.211	0.942–1.556				

HR, hazard ratio; BMI, body mass index.

**Table 5:** Univariate and multivariate analyses of the risk factors for disease-free survival.

	Univariate			Multivariate		
	HRHR	95% CI	*p*	HR	95% CI	*p*
**Sex**			0.078			
Male	1					
Female	1.270	0.973–1.656				
**Age, years**			0.322			
<60	1					
≥60	1.135	0.884–1.458				
**BMI, kg/m^2^**			0.619			
<24	1					
≥24	1.072	0.814–1.413				
**Tumor size, cm**			<0.001			
<5	1					
≥5	1.627	1.264–2.094				
**Histology**			0.013			
Differentiated	1					
Undifferentiated	1.416	1.077–1.861				
**Metastatic lymph node**			<0.001			
<3	1					
≥3	2.756	2.068–3.673				
**Pathologic T stage**			<0.001			0.005
T2	1					
T3	1.512	0.817–2.798	0.188	0.531	0.255–1.106	0.091
T4a	3.386	1.967–5.830	<0.001	0.991	0.486–2.023	0.981
T4b	9.486	3.616–24.881	<0.001	1.883	0.636–5.569	0.253
**Pathologic N stage**			<0.001			
N0	1					
N1	2.234	1.361–3.668	0.001			
N2	3.944	2.463–6.314	<0.001			
N3	5.041	3.203–7.935	<0.001			
**Pathologic TNM stage**			<0.001			<0.001
IB	1					
IIA	1.904	0.586–6.183	0.284	2.382	0.675–8.411	0.177
IIB	2.367	0.826–6.782	0.109	2.539	0.759–8.500	0.131
IIIA	4.299	1.543–11.982	0.005	4.712	1.395–15.913	0.013
IIIB	6.398	2.337–17.515	<0.001	7.287	2.137–24.853	0.002
IIIC	10.316	3.785–28.113	<0.001	10.321	3.016–35.317	<0.001
**Retrieved lymph node**			0.055			
<28	1					
≥28	1.288	0.994–1.668				
**Surgical approach**			0.123			
Open	1					
Laparoscopic	1.218	0.948–1.565				

HR, hazard ratio; BMI, body mass index.

## Discussion

A multicenter prospective Korean trial (KLASS-03) has established the feasibility and safety of LTG in EGC (6). Similarly, in a multicenter trial from China (CLASS-02), in which 214 patients with clinical stage I gastric cancer underwent LTG or OTG, the authors found that the morbidity and mortality of LTG were similar to those of OTG (7). Further, LTG was associated with longer surgical time but less intraoperative bleeding. Additional follow-up is necessary for the establishment of oncological safety of LTG in EGC. The efficacy and safety of LTG for AGC have not yet been proven by multicenter RCTs. Two RCTs, KLASS-06 (NCT03385018) and CLASS-07 (NCT04710758), are still recruiting. As far as our information goes, this is the first multicenter cohort study reporting both the surgical and oncological outcomes of LTG versus OTG in the field of AGC.

LTG is riskier and technically more demanding than laparoscopic distal gastrectomy considering the requirement of complex esophagojejunostomy, which has not yet been standardized, and high expertise needed for lymphadenectomy around the distal pancreas as well as splenic hilum, where the vascular structure can be difficult to visualize and there is a high risk of pancreatic injury. Consequently, there are concerns about postoperative complication rates and mortality rates. Postoperative complications following total gastrectomy are primarily anastomotic leak (21, 22), accounting for 5% to 7% of all cases (23–25). One case series reported a higher perioperative mortality rate and a greater risk of an anastomotic leak following LTG than OTG for EGC (26). For AGC, two retrospective multicenter cohort studies based on Japanese nationwide databases showed that patients who underwent LTG were at a higher risk of anastomotic leak (16, 17). In this study, the mortality rate and incidence of anastomosis-related complications (anastomotic leak, anastomotic bleed, and anastomotic stenosis), however, were similar in both groups. Furthermore, it was shown that LTG could lead to a significant reduction of risk of postoperative complications. Also, LTG led to a lower incidence of intra-abdominal abscess, although LTG had no significant difference in major surgical complications from that of OTG, possibly because the range of gastrectomy and lymphadenectomy with LTG was identical to that with OTG. These findings are in line with a previous meta-analysis that compared LTG with OTG for both EGC and AGC and also suggested that such could be also partly explained by surgeons’ experience (27). Concerning pancreatic complications (e.g., pancreatic fistula, pancreatitis, and pancreatic leakage), the risks could not be analyzed in this study due to the lack of detailed enough data on postoperative complications. According to a systematic review and meta-analysis conducted by Guerra et al. (28), the incidence of pancreatic complications after gastrectomy was estimated to be more than 1%, and minimally invasive surgery posed a higher risk of overall pancreatic complications than open surgery. However, the results from several retrospective cohort studies consistently suggested that there were no differences in the incidence of pancreatic fistula or pancreatitis between LTG and OTG for AGC (14–16, 29), which remain to be further verified by large RCTs. As a complex minimally invasive procedure, LTG, particularly the D2 lymphadenectomy and reconstruction portions of the operation, has a steep learning curve. Studies from Eastern countries estimated that surgeons would require at least over 100 cases of LTG to become proficient (30). Although there were no specific criteria for participating centers and surgeons in this study, all the institutions are high-volume centers where the surgeons are well-experienced in both LTG and OTG.

Several surgical outcomes favored LTG over OTG for AGC, which agreed with the findings of Oh et al. (27). An important advantage of LTG over OTG is the significantly lower amount of perioperative blood loss. With advanced laparoscopic surgical instruments, surgeons could perform fine dissection and meticulous hemostasis under a magnified operative view so that unexpected bleeding and excessive disruptions could be effectively avoided. In addition, a longer proximal resection margin could be achieved in the LTG group. LTG was also shown to significantly shorten the postoperative hospitalization, probably contributed by its minimally invasive nature, lower rate of overall postoperative complications, and lower intraoperative blood loss.

Based on the study findings, we hypothesized that the surgical time of the LTG group was significantly longer for the following reasons: first, LTG deprives the surgeons of the depth of perception, dexterity, tactile feedback, and the straightforward hand–eye coordination that they are accustomed to during conventional OTG, therefore requiring slower and more meticulous operative maneuver (31). Second, the application of the no-touch principle for laparoscopic lymph node dissection at a deep lymph node station during the delicate D2 lymphadenectomy is also a delicate challenge under a narrow surgical field, which is also time-consuming. Third, extra surgical time can also be due to the frequent changing of laparoscopic instruments and the cleaning time needed of the laparoscopic camera. However, despite operation time lengthening, patients’ recovery is faster, and incisions are cosmetically more acceptable and has a lesser risk of postoperative complications (which could take weeks to recover) under laparoscopy than the traditional open approach, thereby contributing a higher benefit-to-risk ratio.

Some surgeons remain prudent to routinely perform LTG for EGC, and more surgeons remain skeptical about the oncological adequacy of LTG in treating AGC, with some even reluctant to perform LTG for AGC. The number of harvested lymph nodes and the extent of lymphadenectomy, on which long-term survival depends, were taken into account in this study. We observed that the difference in the number of retrieved lymph nodes was insignificant between LTG and OTG, and LTG was not significantly different from OTG in the percentage of cases with at least 15 lymph nodes harvested. Hence, for both LTG and OTG, radical D2 lymphadenectomy could be equally achieved, thereby contributing to both 5-year DFS and 5-year OS rates being comparable for both approaches. Moreover, multivariate analyses corroborated that the surgical approach did not affect DFS or OS among AGC patients. Once clear margins and complete lymphadenectomy could be strictly ensured, patients’ long-term survival primarily depended on the biological characteristics of cancer rather than the choice of techniques (32).

There are still several limitations in the current study worth mentioning. First, selection bias could have existed due to the retrospective nature of this cohort. Second, although PSM was used to reduce such bias, tumor size, tumor histology, and pT stage were still not similarly distributed between the two procedures. Lastly, due to lack of related data, we failed to include several other important factors into the analyses, which might have affected surgical and oncological outcomes, i.e., comorbidities of patients, time of first LTG in each center, and learning curve of the surgeons. Despite these limitations, this multicenter cohort study offers a high level of clinical evidence for the feasibility and safety of LTG over OTG, but the findings remain to be confirmed by future large-scale, prospective RCTs such as KLASS-06 (NCT03385018) and CLASS-07 (NCT04710758).

## Conclusions

The results of this study showed the non-inferiority of LTG with D2 lymphadenectomy on the oncological safety to OTG and its superiority over OTG on the surgical outcomes, including fewer postoperative complications, less intraoperative bleeding, and shorter postoperative hospital stay. LTG with D2 lymphadenectomy could be a potential valid treatment for patients with AGC when it is carried out by well-trained surgeons with adequate experience at large hospitals.

## Data Availability Statement

The raw data supporting the conclusions of this article will be made available by the authors, without undue reservation.

## Ethics Statement

The studies involving human participants were reviewed and approved by the Ethics Review Committee of Guangdong Provincial People’s Hospital and Guangdong Academy of Medical Sciences; Ethics Review Committee of Fujian Cancer Hospital and Fujian Medical University Cancer Hospital; and Ethics Review Committee of Guangdong Provincial Hospital of Chinese Medicine and the Second Affiliated Hospital of Guangzhou University of Chinese Medicine. Written informed consent for participation was not required for this study in accordance with the national legislation and the institutional requirements.

## Author Contributions

XF, XC, and ZY have designed the study, analyzed the data, prepared the figures and tables, and written the manuscript. JW, LC, and YL have conceived the study, supervised the results, critically revised the manuscript, and were responsible for the financial support and the corresponding works. WX, XY, and WW have critically revised the manuscript. XF, XC, and ZY have contributed equally to this work and share the first authorship. All authors contributed to the article and approved the submitted version.

## Funding

This work was supported by grants of the Science and Technology Plan of Guangzhou, Guangdong Province, China (grant number: 202102080230), the Medical Scientific Research Foundation of Guangdong Province, China (grant number: A2020019), and the Natural Science Foundation of Guangdong Province, China (grant number: 2020A1515010573).

## Conflict of Interest

The authors declare that the research was conducted in the absence of any commercial or financial relationships that could be construed as a potential conflict of interest.

## Publisher’s Note

All claims expressed in this article are solely those of the authors and do not necessarily represent those of their affiliated organizations, or those of the publisher, the editors and the reviewers. Any product that may be evaluated in this article, or claim that may be made by its manufacturer, is not guaranteed or endorsed by the publisher.
